# Expression of Hexokinase-2 (HK2), Glutaminase-1 (GLS1) and Fatty Acid Synthase (FASN) in Gastric Cancer and Their Prognostic Significance

**DOI:** 10.3390/medsci14010148

**Published:** 2026-03-19

**Authors:** Elisa García-Martínez, Leonardo S. Lino-Silva, Adriana Romo-Pérez, Leticia Bornstein-Quevedo, Alma Chavez-Blanco, Guadalupe Dominguez-Gomez, Horacio N. Lopez-Basave, Alejandro Padilla-Rosciano, Consuelo Diaz-Romero, Aurora Gonzalez-Fierro, Alfonso Duenas-Gonzalez

**Affiliations:** 1 Basic Research Subdirection, National Cancer Institute of Mexico (INCan), Mexico City 14080, Mexico; elisagm1994@hotmail.com (E.G.-M.); celular_alma@hotmail.com (A.C.-B.); dguadalupeisabel@yahoo.com.mx (G.D.-G.); aufierro@hotmail.com (A.G.-F.); 2 Department of Pathology, National Cancer Institute of Mexico (INCan), Mexico City 14080, Mexico; saul.lino.sil@gmail.com; 3 Institute of Chemistry, National Autonomous University of Mexico, Mexico City 04510, Mexico; adriana.romo@iquimica.unam.mx; 4 InmunoQ, Laboratorio de Patología, Inmunohistoquímica y Biología Molecular, Mexico City 03100, Mexico; leticia.bornstein@inmunoq.org; 5 Department of Gastroenterology, National Cancer Institute of Mexico (INCan), Mexico City 14080, Mexico; hlopezb@incan.edu.mx (H.N.L.-B.); alejandropadilla@prodigy.net.mx (A.P.-R.); 6 Department of Medical Oncology, National Cancer Institute of Mexico (INCan), Mexico City 14080, Mexico; onco_diazz28@yahoo.com.mx; 7 Direction of Teaching and Research, Maternal and Child Institute of the State of Mexico (IMIEM), Toluca 50170, Mexico

**Keywords:** gastric cancer, hexokinase-2, glutaminase-1, fatty acid synthase

## Abstract

**Background/Objectives:** To evaluate the immunohistochemical expression of hexokinase-2 (HK2), glutaminase-1 (GLS1), and fatty acid synthase (FASN) and its prognostic significance in diffuse gastric adenocarcinoma. **Materials and Methods:** Formalin-fixed paraffin-embedded tissue samples from 92 patients with diffuse gastric adenocarcinoma were analyzed. Immunohistochemistry (IHC) was performed to assess the expression of HK2, GLS1 and FASN. Expression levels were evaluated semi-quantitatively based on staining intensity and the percentage of positive cells. Associations between enzyme expression and clinicopathological features were assessed using the Chi-square test. Kaplan–Meier survival analysis was employed to evaluate progression-free survival (PFS) and overall survival (OS) and the log-rank test and Cox proportional hazards models were used for statistical analysis. **Results:** HK2 and FASN were overexpressed in 20.7% and 22.8% of patients, respectively, and were significantly associated with advanced tumor stage. In contrast, GLS1 expression, found in 30.4% of patients, did not independently correlate with clinicopathological characteristics. Furthermore, HK2 expression and co-expression of HK2/FASN (10.9%) and HK2/GLS1/FASN (8.7%) were associated with progressive disease. In the univariate analysis, stage, HK2 overexpression, and co-expression of HK2/FASN and HK2/GLS1/FASN were associated with shorter survival. However, only stage retained prognostic value in the multivariate analysis. **Conclusions:** Co-expression of these key metabolic enzymes remains a promising candidate as prognostic markers and therapeutic targets. Concurrent targeting of these metabolic pathways may offer novel therapeutic opportunities for patients with advanced-stage gastric cancer.

## 1. Introduction

Gastric cancer (GC) remains a challenging global health issue, ranking among the top five causes of cancer-related deaths. According to recent global cancer statistics, there were approximately 968,784 new cases and 660,175 deaths from GC in 2022 [1]. The prognosis for GC is typically poor with a five-year survival rate of about 6% for advanced stage disease [2].

Biomarkers have become pivotal in the comprehensive management of gastric cancer serving multiple critical roles including early detection, accurate diagnosis, monitoring of treatment efficacy and informing therapeutic strategies. Among the biomarkers frequently employed in gastric cancer management, HER2, PD-L1 and microsatellite instability (MSI) stand out for their significance, each offering valuable insights into prognosis and guiding treatment approaches [3]. For example, in HER2-positive patients, the combination of trastuzumab with chemotherapy as a first-line treatment results in a median overall survival (mOS) extension of 2.7 months compared to chemotherapy alone. Additionally, trastuzumab deruxtecan, when used as a second-line treatment in patients who have progressed following trastuzumab therapy, increases mOS from 8.4 to 12.5 months compared to single-agent chemotherapy [4,5]. These findings are promising, underscoring the need for continued exploration and development of novel therapeutic alternatives.

There is currently a renewed interest in studying tumor metabolism due to its potential to update the development of drugs that can target specific aspects of the tumor metabolic phenotype [6]. Malignant cells heavily depend on two critical nutrients, glucose and glutamine for macromolecule synthesis, energy production, the generation of reducing equivalents and the maintenance of redox balance [7]. Additionally, malignant cells preferentially synthesize fatty acids (FA) de novo rather than acquiring them from circulation [8]. In the process of de novo FA synthesis, both glucose and glutamine provide citrate, which is essential for producing acetyl-CoA and NADPH required for FA synthesis [9]. Thus, glycolysis, glutaminolysis and FA synthesis represent a shared metabolic pathway network in cancer cells [10]. Notably, these three pathways have been shown to be active in gastric cancer [11]. Correspondingly, the genes encoding enzymes involved in these pathways—hexokinase-2 (HK2) for glycolysis, glutaminase-1 (GLS1) for glutaminolysis and fatty acid synthase (FASN) for FA synthesis—are reported to be overexpressed in malignant cell lines, including those derived from gastric cancer [12]. The overexpression of these enzymes has been observed in both cell lines and primary tumors, and this overexpression is associated with aggressive clinicopathological features and poor prognosis [13]. However, to our knowledge, the simultaneous evaluation of the expression of these three critical metabolic enzymes—HK2, GLS1 and FASN—has not yet been conducted in gastric cancer.

## 2. Materials and Methods

### 2.1. Patients

This retrospective study, conducted between 2022 and 2023, utilized clinical data collected from 2005 to 2016 from the database the Instituto Nacional de Cancerología, Mexico. The inclusion criteria comprised patients with histological diagnosis of diffuse gastric adenocarcinoma (DGC) submitted to curative or palliative gastrectomy and had clinical follow-up data and their formalin-fixed paraffin-embedded tissues sections available. The clinical data were collected from the Institutional electronic file system. Progression-Free Survival (PFS) was considered from the date of diagnosis to the date of relapse or progression. Overall survival (OS) was defined by the interval from diagnosis to death from any cause.

### 2.2. Ethical Issues

The study was approved by the Institutional Ethics committee (Approval number: (018/047/IBI) (CEI/1313/18). This study which involves human participants is following the 1964 Helsinki declaration and its later amendments.

### 2.3. Immunohistochemical Analysis

Formalin-fixed, paraffin-embedded (FFPE) tissue blocks from patients diagnosed with diffuse gastric cancer were retrieved from the archives of the Pathology Department. Histopathological diagnoses were confirmed by two board-certified pathologists (L-S, B-Q), based on the most recent World Health Organization (WHO) classification of gastric adenocarcinoma. The TNM staging for each case was determined according to the guidelines provided in the 8th edition of the Union for International Cancer Control (UICC). For TMA core selection, representative areas of malignant tissue were selected from H&E slides by two independent board-certified pathologists, prioritizing regions with high tumor cellularity (>70%) and minimal necrosis. From the corresponding tumor blocks, a 5 mm tissue core was extracted and incorporated into a tissue microarray (TMA). Each TMA block contained 14 to 15 individual tumor samples. Tissue sections, 4 µm in thickness, were then cut from the paraffin blocks and prepared for further analysis.

### 2.4. Immunohistochemistry (IHC)

The immunohistochemistry was performed on the Ventana Benchmark ULTRA platform (Ventana Medical Systems, Inc., Tucson, AZ, USA) using the following primary antibodies: Anti-Hexokinase-2 (HK2) Rabbit Monoclonal Antibody (Cell Signaling: C64G5) at a 1:50 dilution; Anti-Fatty Acid Synthase (FASN) Mouse Monoclonal Antibody (Santa Cruz Biotechnology, Inc.: Dallas, TX, USA: sc-48357) at a 1:200 dilution; and Anti-Glutaminase (GLS1) Rabbit Polyclonal Antibody (Abcam: Cambridge, UK: ab260047) at a 1:1200 dilution. The immunoreactivity for each antibody in neoplastic cells was evaluated semi-quantitatively. A scoring system based on staining intensity (0: negative, 1: weak, 2: moderate, 3: strong) was multiplied by the percentage of positive cells within the tissue, resulting in a final score ranging from 0 to 300. IHC scoring was performed independently by two pathologists (L-S, B-Q) blinded to clinical data, with discrepancies resolved by consensus. Cut-off values were selected based on established literature thresholds for these enzymes and the distribution of scores in our samples, aiming to distinguish biologically meaningful overexpression. No formal ROC or sensitivity analysis was performed. The final scores were interpreted as follows: for HK2, scores of 0–180 were considered negative and >180 positive; for GLS1, 0–150 were negative and >150 positive; and for FASN, 0–210 were negative and >210 positive.

### 2.5. Statistical Analysis

The association between FASN, HK2, and GLS1 expression and clinicopathological characteristics was analyzed using the Chi-square test. Kaplan–Meier analysis was employed to assess the prognostic significance of PFS and OS. Survival distributions were evaluated using the log-rank test. A stepwise Cox proportional hazards model was used to conduct univariate and multivariate survival analyses. A *p*-value of less than 0.05 was considered statistically significant.

## 3. Results

The primary clinical characteristics of the 92 patients are summarized in Table 1. The median age was 55 years (range: 18–74) and 52 patients (56.5%) were female. A significant portion of the patients were diagnosed at stage III (30.4%) or stage IV (42.4%). Of the total cohort, 54 patients (58.7%) underwent R0 surgical resection, and the majority received either adjuvant or neoadjuvant therapy (Table 1). The regimen of 5-Fluorouracil/cisplatin, with or without epirubicin, was utilized in both the neoadjuvant and adjuvant settings. The median follow-up period for the entire group was 31.8 months (range: 2–116).

**Figure 1:**
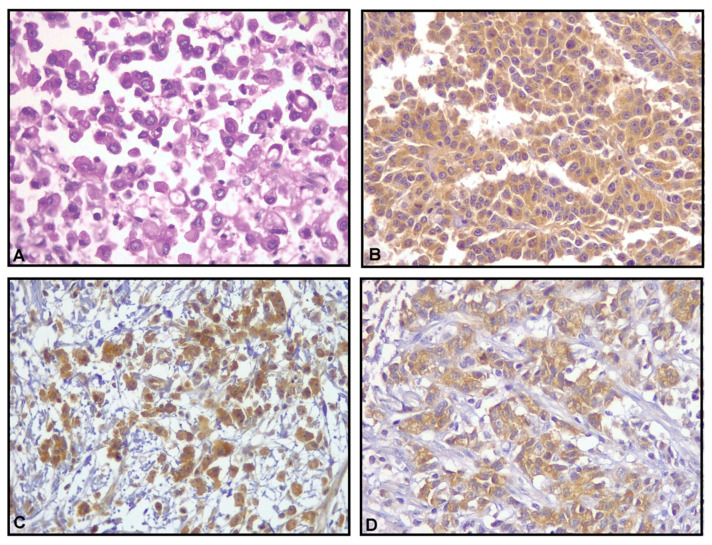
Immunohistochemical detection of hexokinase-2 (HK2), glutaminase-1 (GLS1), and fatty acid synthase (FASN). Images correspond to cross-sections of gastric cancer. (**A**) Poorly differentiated diffuse gastric adenocarcinoma (H&E; 40×). (**B**) HK2-positive cytoplasmic staining in diffuse gastric adenocarcinoma (40×). (**C**) GLS1-positive nuclear and cytoplasmic staining (40×). (**D**) FASN-positive cytoplasmic staining in diffuse gastric adenocarcinoma (40×).

As shown in Table 2, diffuse gastric adenocarcinoma exhibited varying levels of overexpression of these enzymes, either individually or in combination. HK2 was positively expressed in 19 patients (20.7%), while 73 patients (79.3%) were negative for this enzyme. GLS1 was expressed in 28 patients (30.4%), with 64 patients (69.6%) lacking expression. FASN expression was positive in 21 patients (22.8%) and negative in 71 patients (77.2%). Combined expression analysis revealed that 10 patients (10.9%) co-expressed HK2 and FASN, while 8 patients (8.7%) co-expressed all three enzymes (HK2, GLS1, and FASN). Notably, only one patient co-expressed both GLS1 and HK2, and none co-expressed GLS1 and FASN (Figure 1).

We evaluated whether the overexpression of these metabolic enzymes correlated with clinicopathological characteristics, and the results are shown in Table 3. Notably, the overexpression of HK2, FASN, HK2/FASN, and HK2/GLS1/FASN—but not GLS1 alone—was significantly associated with tumor stage, with most expressing patients classified as stage III. Additionally, overexpression of these enzymes, except for GLS1, was linked to R1 resection, likely due to the association with more advanced stages. Overexpression of HK2, HK2/FASN, and HK2/GLS1/FASN was also associated with disease progression and HK2 overexpression was correlated with mortality. All patients co-overexpressing HK2/FASN (n = 10) or HK2/GLS1/FASN (n = 8) died, with these findings approaching statistical significance. Due to the lack of correlation between GLS1 expression and any clinicopathological variables, no further analysis was performed on GLS1 combinations with HK2 or FASN. However, despite GLS1’s individual lack of prognostic significance, its inclusion was retained in the analysis of the simultaneous expression of all three enzymes. Notably, there was no significant association between enzyme expression and chemotherapy use.

**Figure 2:**
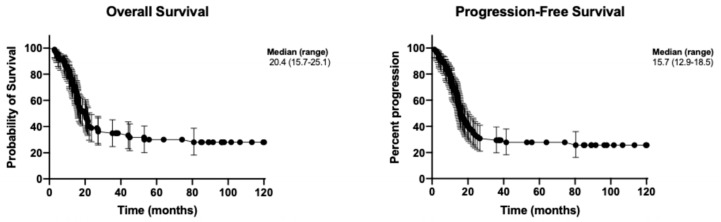
Overall Survival and Progression-Free Survival.

**Figure 3:**
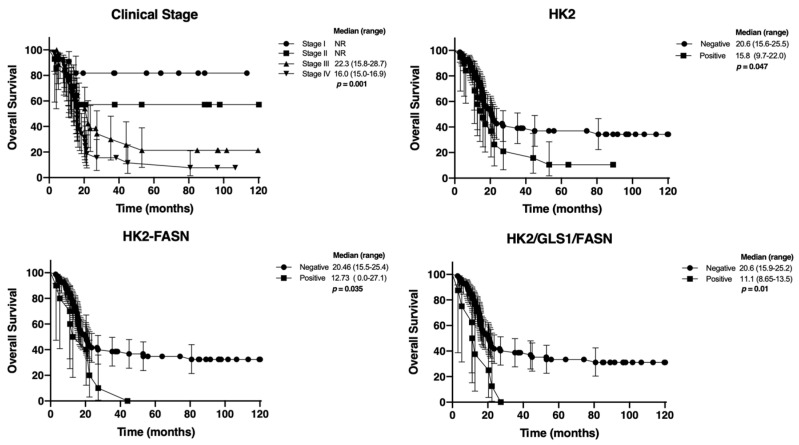
Univariate analysis of Overall Survival. Clinical stage was the most significant prognostic factor for OS.

All 92 patients completed clinical follow-up data. At a median follow-up time of 31.8 months (range: 2–116 months), the median progression-free survival (PFS) and overall survival (OS) were 15.7 months (range: 12.9–18.5 months) and 20.4 months (range: 15.7–25.1 months), respectively (Figure 2). Univariate analysis revealed that only disease stage had prognostic significance for PFS, with the median PFS not reached for stages I–II, while it was 17.6 months and 14.1 months for stages III and IV, respectively (*p* = 0.0001, Table 4). Although the overexpression of HK2, HK2/FASN, and HK2/GLS1/FASN showed a trend toward shorter PFS, these findings were not statistically significant. For OS, disease stage remained a significant poor prognostic factor, with the median OS not reached for stages I–II, and 23.3 months and 16 months for stages III and IV, respectively (*p* = 0.001, Figure 3). Among the enzyme expressions analyzed, HK2, HK2/FASN and HK2/GLS1/FASN were significantly associated with shorter OS in the univariate analysis (Figure 3). However, multivariate analysis for OS (Table 5) demonstrated that the only independent variable determining prognosis was disease stage, with a Hazard Ratio of 2.0057 (95% CI: 1.4319–2.8095), *p* = 0.0001.

## 4. Discussion

The findings of this study demonstrate that diffuse gastric adenocarcinoma exhibits overexpression of key metabolic enzymes HK2, GLS1, and FASN determined by immunohistochemistry. These enzymes are critical drivers of glycolysis, glutaminolysis and de novo fatty acid synthesis, metabolic pathways that are frequently upregulated in gastric cancer [14,15]. Notably, the overexpression of HK2 as well as the combined expression of HK2/FASN and HK2/GLS1/FASN, was associated with a trend toward shorter PFS and with shorter OS in univariate analysis; however, multivariate models indicate that these markers are not independent predictors, likely confounded by their strong association with advanced disease stage. Thus, their utility may lie more in complementing stage-based prognostication rather than serving as standalone biomarkers and their prognostic significance must be interpreted cautiously, given their low overall prevalence and strong enrichment in advanced stages. This suggests these markers may primarily reflect aggressive disease biology rather than independently driving outcomes. Yet, our findings are noteworthy, as prior studies on primary gastric adenocarcinoma have generally evaluated protein or mRNA expression of these enzymes individually rather than simultaneously [13], highlighting the potential therapeutic relevance of targeting multiple pathways concurrently despite us acknowledging the potential limitations in clinical translation due to low concurrent expression prevalence, even in advanced stages, yet patient selection via IHC profiling could enrich for responders.

Hexokinase enzymes catalyze the phosphorylation of glucose to glucose-6-phosphate, the first step in glycolysis. Among the four hexokinase isoforms (HK1–4), HK2 is specifically implicated in the Warburg effect, a hallmark of cancer metabolism. HK2 translocate to the outer mitochondrial membrane, where it interacts with the voltage-dependent anion channel (VDAC), inhibiting apoptosis mediated by mitochondria [16]. Although HK2 has been studied in gastric adenocarcinoma, results vary across studies. Rho et al. found HK2 overexpression in 16.7% of gastric cancers (43 out of 257 cases), which correlated with Bcl-2 expression and was linked to reduced survival [17]. Another study observed HK2 overexpression in 21.3% of samples (40 out of 188), where it was identified as an independent adverse prognostic factor [18]. Conversely, a third study reported HK2 overexpression in only 4.1% of cases (7 out of 152), with no correlation to clinicopathological features or prognosis [19].

Fatty acid synthase (FASN) is a central regulator of lipid metabolism, playing a critical role in tumorigenesis, including in gastric cancer [20]. In one study, FASN positivity was observed in 28.9% of patients and was associated with decreased three-year overall survival (OS) [21]. Another study reported FASN overexpression in 50.8% of tumors and showed a correlation between intense FASN expression and shorter survival times [22].

**Figure 4:**
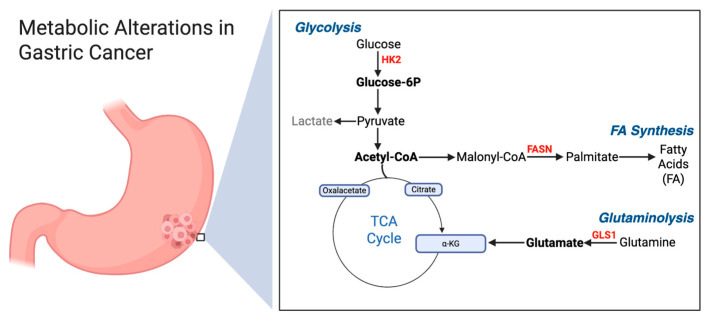
Overexpression of enzymes involved in key steps of glycolysis (HK2), glutaminolysis (GLS1), and fatty acid synthesis (FASN) in GC. The increase in HK2 results in more pyruvate production and ultimately more acetyl-CoA, which can enter the TCA cycle to generate energy or be used as fuel for fatty acid synthesis through the conversion of malonyl-CoA to palmitate by the FASN enzyme. Furthermore, increased glutamate production by GLS1 can be converted to α-KG (α-ketoglutarate), which continues generating energy via the TCA cycle.

GLS1, a key enzyme in glutaminolysis, has been less studied in gastric cancer. There is only a single study on GLS1 expression in primary gastric cancers [23]. Jiang et al. reported GLS1 overexpression in 75.6% of gastric tumors compared to 19.1% in adjacent tissues, and its expression was associated with larger tumor size and lymph node metastasis, although its prognostic significance was not determined [24]. Figure 4 summarizes how the overexpression of the enzymes HK2, GLS1 and FASN is related to gastric cancer metabolism.

Our findings on enzyme overexpression align with previous studies, though the percentage of expression varies. Factors such as patient population differences, antibodies, tissue processing techniques, and semi-quantitative scoring methods likely account for these discrepancies. However, these enzymes are overexpressed in gastric adenocarcinoma. Preclinical studies have demonstrated that genetic depletion or pharmacological inhibition of HK2, GLS1, and FASN induces antitumor effects in gastric cancer cell lines. For example, HK2 depletion via miR-181b overexpression suppresses proliferation and migration in NCI-N87 and MGC80-3 gastric cancer cells [25]. Similarly, Licochalcone A, a traditional Chinese medicine, reduces HK2 expression and induces apoptosis in vitro and in vivo by inhibiting glycolysis, with HK2 overexpression reversing these effects [26]. Furthermore, the glycolysis inhibitor 2-deoxy-d-glucose, alongside oleanolic acid, exerts antitumor effects in MKN-45 and SGC-7901 cells [27].

Regarding fatty acid synthesis, FASN knockdown in SGC-7901 cells inhibited proliferation and reversed epithelial-to-mesenchymal transition (EMT) via the AMPK/mTOR and AKT/mTOR signaling pathways [28,29]. GLS1, overexpressed in tumors with high glutaminolysis rates, has also been shown to induce antitumor effects upon genetic or pharmacological depletion in gastric cancer models [30]. Previous work in the AGS gastric cancer cell line demonstrated that the combined use of HK2, GLS1, and FASN inhibitors (lonidamine, DON, and orlistat, respectively) produced significant antitumor effects in vitro and was well-tolerated in vivo [12,31].

Our findings indicate that approximately 10% of patients exhibit simultaneous overexpression of HK2/FASN or HK2/GLS1/FASN, suggesting that gastric adenocarcinomas may preferentially rely on single or combined metabolic pathways. This supports the idea that metabolic reprogramming can shift upon inhibition of specific pathways. For example, inhibiting both glycolysis and fatty acid synthesis enhances the effect of the FASN inhibitor orlistat in gastric cancer cells [32]. Beyond therapeutic potential, these metabolic enzymes could serve as biomarkers for early detection and prognostication. GLS1 expression in gastric cancer tissue has been shown to have sufficient sensitivity and specificity for detecting early-stage gastric cancer [24], while metabolic profiling of glycolysis and lipid pathways could help identify gastric cancer subtypes and inform therapy selection [33].

While our study provides important insights into the prognostic role of HK2, GLS1, and FASN in diffuse gastric adenocarcinoma, it is subject to several limitations that should be considered. First, the sample size was relatively small, which may have reduced statistical power, potentially contributing to the loss of significance in multivariate models. Second, we employed a semi-quantitative IHC scoring system, which, while standard, introduces subjectivity and relies on visual assessment. Third, the study focused exclusively on diffuse-type gastric adenocarcinoma from a single institution, limiting generalizability to other histological subtypes which may exhibit different metabolic profiles. Additionally, cut-off values for enzyme positivity were selected based on literature precedents and score distributions rather than optimized through formal sensitivity analyses like receiver operating characteristic (ROC) curves, which could refine threshold selection in future validations. The retrospective design also introduces potential selection biases, and we did not correlate enzyme expression with other established biomarkers (e.g., HER2, PD-L1, or MSI status) or functional assays to confirm mechanistic roles. Moreover, treatment heterogeneity (e.g., varying neoadjuvant/adjuvant regimens) and the median follow-up of 31.8 months may not fully capture long-term outcomes. These factors underscore the need for larger, prospective, multi-center cohorts that encompass diverse histological subtypes, incorporate advanced statistical optimizations, and integrate molecular and functional validations to confirm and extend our findings. Moreover, the mechanistic role of enzyme overexpression in prognosis was not studied. Nevertheless, this is the first study to demonstrate simultaneous overexpression of HK2, GLS1, and FASN in primary gastric adenocarcinoma, warranting further investigation. Targeting these metabolic pathways could provide therapeutic benefit [34], as demonstrated by our previous findings that the combination of HK2, GLS1, and FASN inhibitors is both effective and well-tolerated in vivo [12,31,35].

## 5. Conclusions

In conclusion, further studies needed to elucidate the clinical relevance of the concurrent overexpression of key glycolysis, glutaminolysis and fatty acid synthesis enzymes in gastric adenocarcinoma, as their pharmacological targeting may have therapeutic potential.

## Figures and Tables

**Table 1:** Patient characteristics.

Age (Median)	55 (18–74)
**Follow-up (months)—median**	31.8 months (2–116)
**Sex—n (%)**	
Female	52 (56.5%)
Male	40 (43.5%)
**Clinical Stage—n (%)**	
I	11 (12%)
II	14 (15.2%)
III	28 (30.4%)
IV	39 (42.4%)
**Residual tumor (R) classification**	
R0	54 (58.7%)
R1	38 (41.3%)
**Neoadjuvant therapy—n (%) ^a^**	
No	77 (83.7%)
Yes	15 (16.3%)
**Adjuvant therapy—n (%) ^b^**	
No	25 (27.2%)
Yes	67 (72.8%)
**Cancer Progression—n (%)**	
No	31 (33.7%)
Yes	61 (66.3%)
**Death—n (%)**	
No	34 (37%)
Yes	58 (63%)

^a^ Neoadjuvant therapy comprised 5-Fluouracil/Cisplatin (70%) and 5-Fluouracil/Cisplatin/Epirubicin (30%) of patients. ^b^ Adjuvant therapy comprised 5-Fluouracil/Cisplatin.

**Table 2:** Immunohistochemical enzyme expression.

Enzyme Expression	HK2 Pos > 180 Neg < 180	GLS1 Pos > 150 Neg < 150	FASN Pos > 210 Neg < 210	HK2/FASN	HK2/GLS1/FASN
Positive (%)	19 (20.7%)	28 (30.4%)	21 (22.8%)	10 (10.9%)	8 (8.7%)
Negative (%)	73 (79.3%)	64 (69.6%)	71 (77.2%)	82 (89.1%)	84 (91.3%)
Total	92	92	92	92	92

Abbreviations: HK2—hexokinase-2, GLS1—glutaminase-1, FASN—fatty acid synthase.

**Table 3:** Clinicopathological characteristics and enzyme expression.

		HK2			FASN			GLS1			HK2/FASN			HK2/FASN/GLS1			TOTAL (%)
		0–180 n (%)	>180 n (%)	*p*	0–210 n (%)	>210 n (%)	*p*	Negative n (%)	Positive n (%)	*p*	Negative n (%)	Positive n (%)	*p*	Negative n (%)	Positive n (%)	*p*	
**Sex**	**Male**	35 (47.9)	5 (26.3)	0.09	29 (40.8)	11 (52.4)	0.3	25 (39.1)	15 (53.6)	0.1	37 (45.1)	3 (30)	0.362	47 (56)	5 (62.5)	0.7	40 (43.5)
	**Female**	38 (52.1)	14 (73.7)		42 (59.2)	10 (47.6)		39 (60.9)	13 (46.4)		45 (54.9)	7 (70)		37 (44)	3 (37.5)		52 (56.5)
**Age—Me** **an**		54.7 (SD 13.943)	50.32 (SD 13.288)	0.858	54.76 (SD 13.874)	50.52 (SD 13.6)	0.9	54.13 (SD 14.023)	53.04 (SD 13.680)	0.8	54.62 (SD 13.792)	47 (SD 13.081)	0.6	54.29 (SD 13.813)	48.63 (SD 14.111)	0.9	
**Clinical** **Stage**	**I**	9 (12.3)	2 (10.5)	0.031 *	9 (12.7)	2 (9.5)	0.002 *	7 (10.9)	4 (14.3)	0.9	11 (13.4)	0	0.004 *	11 (13.1)	0	0.034 *	11 (12)
	**II**	12 (16.4)	2 (10.5)		11 (15.5)	3 (14.3)		10 (15.6)	4 (14.3)		13 (15.9)	1 (10)		13 (15.5)	1 (12.5)		14 (15.2)
	**III**	17 (23.3)	11 (57.9)		15 (21.1)	13 (61.9)		15 (23.4)	13 (46.4)		20 (24.4)	8 (80)		22 (26.2)	6 (75)		28 (30.4)
	**IV**	35 (47.9)	4 (21.1)		36 (50.7)	3 (14.3)		32 (50)	7 (25)		38 (46.3)	1 (10)		38 (45.2)	1 (12.5)		39 (42.4)
**(R) Classif.**	**R0**	34 (46.6)	4 (21.1)	0.044 *	36 (50.7)	3 (14.3)	0.004 *	29 (45.3)	8 (28.6)	0.168	37 (45.1)	0	0.006 *	37 (44)	0	0.015 *	37 (40.2)
	**R1**	39 (53.4)	15 (78.9)		35 (49.3)	18 (85.7)		35 (54.7)	20 (71.4)		45 (54.9)	10 (100)		47 (56)	8 (100)		55 (59.8)
**BMI**	**Normal**	28 (38.4)	9 (47.4)	0.496	30 (42.3)	7 (33.3)	0.5	27 (42.2)	10 (35.7)	0.812	32 (39)	5 (50)	0.6	34 (40.5)	3 (37.5)	0.7	37 (40.2)
	**OW**	30 (41.1)	5 (26.3)		25 (35.2)	10 (47.6)		24 (37.5)	11 (39.3)		31 (37.8)	4 (40)		31 (36.9)	4 (50)		35 (38)
	**Obese**	15 (20.5)	5 (26.3)		16 (22.5)	4 (19)		13 (20.3)	7 (25)		19 (23.2)	1 (10)		19 (22.6)	1 (12.5)		20 (21.7)
**PD**	**No**	29 (39.7)	2 (10.5)	0.016 *	27 (38)	4 (19)	0.1	19 (29.7)	12 (42.9)	0.219	31 (37.8)	0	0.017 *	31 (36.9)	0	0.048 *	31 (33.7)
	**Yes**	44 (60.3)	17 (89.5)		44 (62)	17 (81)		45 (70.3)	16 (57.1)		51 (62.2)	10 (100)		53 (63.1)	8 (100)		61 (66.3)
**Death**	**No**	32 (43.8)	2 (10.5)	0.007 *	28 (39.4)	6 (28.6)	0.365	15 (23.4)	7 (25)	0.8	22 (26.8)	0	0.06	22 (26.2)	0	0.09	22 (23.9)
	**Yes**	41 (56.2)	17 (89.5)		43 (73.2)	15 (71.4)		49 (76.6)	21 (75)		60 (73.2)	10 (100)		62 (73.8)	8 (100)		70 (76.1)

Abbreviations: HK2—hexokinase-2, FASN—fatty acid synthase, GLS1—glutaminase-1, SD—Standard Deviation, BMI—Body Mass Index, OW—overweight, Classif.—classification, PD—Progressive Disease. * Statistical significance.

**Table 4:** Univariate analysis of PFS and OS.

Progression-Free Survival			Overall Survival	
Variable	Median (Months)	*p* Value	Median (Months)	*p* Value
Sex—n (%)				
Female	14.9	0.153	17.1	0.087
Male	22.9		23.5	
Clinical Stage—n (%)				
I	NR	0.001	NR	0.001
II	NR		NR	
III	17.6		23.3	
IV	14.1		16.0	
BMI—n (%)				
Normal	15.6	0.646	21.5	0.820
Overweight	15.6		16.6	
Obese	17.6		20.4	
Hexokinase-2				
0–180	17.7	0.089	20.6	0.047
>180	14.9		15.8	
Glutaminase-1				
Negative	15.6	0.343	18.4	0.560
Positive	21.5		22.3	
Fatty Acid Synthase				
0–210	15.7	0.299	20.2	0.419
>210	16.9		20.4	
HK2/FASN				
Negative	15.7	0.070	20.4	0.035
Positive	14.9		12.7	
HK2/GLS1/FASN				
Negative	15.7	0.072	20.6	0.010
Positive	11.1		11.1	

Abbreviations: HK2—hexokinase-2, FASN—fatty acid synthase, GLS1—glutaminase-1.

**Table 5:** Multivariate analysis.

Variable	Overall Survival			
	H.R.	Low 95% CI	High 95% CI	*p* Value
**Clinical Stage**	2.0057	1.4319	2.8095	0.0001
**HK2**	2.0259	0.8905	4.6087	0.0923
**HK2/FASN**	0.6319	0.1298	4.6087	0.5696
**HK2/GLS1/FASN**	2.3839	0.5026	11.3059	0.2740

Abbreviations: H.R.—Hazard Ratio, CI—Confidence Interval, HK2—hexokinase-2, FASN—fatty acid synthase, GLS1—glutaminase-1.

## Data Availability

The original contributions presented in this study are included in the article. Further inquiries can be directed to the corresponding author.
